# Chelerythrine Attenuates the Inflammation of Lipopolysaccharide-Induced Acute Lung Inflammation Through NF-κB Signaling Pathway Mediated by Nrf2

**DOI:** 10.3389/fphar.2018.01047

**Published:** 2018-09-26

**Authors:** Lu Fan, Ye Fan, Li Liu, Weiwei Tao, Xin Shan, Yu Dong, Lin Li, Sen Zhang, Hanqing Wang

**Affiliations:** ^1^School of Medicine and Life Sciences, Nanjing University of Chinese Medicine, Nanjing, China; ^2^Department of Emergency Medicine, Nanjing General Hospital/Jinling Hospital, Medical School of Nanjing University, Nanjing, China; ^3^School of Pharmacy, Guangdong Medical University, Dongguan, China; ^4^Center for Translational Systems Biology and Neuroscience, School of Basic Biomedical Science, Nanjing University of Chinese Medicine, Nanjing, China; ^5^College of Pharmacy, Ningxia Medical University, Yinchuan, China

**Keywords:** chelerythrine, inflammation, LPS, ALI, NF-κB, Nrf2

## Abstract

Chelerythrine (CH), is a kind of benzo[c] phenanthridine alkaloid isolated from plants such as Chelidonium, with pharmacological activities as antitumor, antibiosis and anti-inflammation. However, few studies have demonstrated whether CH could protect against lipopolysaccharide (LPS)-induced acute lung injury (ALI), and the underlying mechanism is also uncertain. The purpose of the present study was to investigate the anti-inflammatory effects of CH on LPS-induced ALI in mice and in RAW264.7 cells. In this study, we demonstrated that treatment with CH significantly ameliorated LPS-induced pathological changes in the lung. CH also attenuated LPS-induced W/D ratio, inflammatory cell infiltration. Meanwhile, LPS-induced Tumor necrosis factor-alpha (TNF-α), interleukin 6 (IL-6), and interleukin 1β (IL-1β) production and oxidative stress were markedly suppressed by CH. Furthermore, western blot showed that CH suppressed LPS-stimulated inflammation of RAW264.7 cells through activation of nuclear factor kappa-B (NF-κB) pathway. Knocking down of nuclear factor erythroid 2-related factor 2 (Nrf2) led to the reduction of nuclear translocation of the NF-κB p65, which triggered inflammation. These experimental results provided evidence that CH could be a potential therapeutic candidate for the intervention of ALI caused by LPS.

## Introduction

Acute lung injury (ALI) is a major cause of morbidity and mortality in intensive care units (Maca et al., 2017). ALI, or its more severe form, acute respiratory distress syndrome (ARDS), is characterized by severe hypoxemia, pulmonary edema, neutrophil accumulation in the lung and intense pulmonary inflammatory response with the overproduction of inflammatory cytokines (Matthay et al., 2017). It is a common clinical problem with a high mortality rate of 30–40% despite significant advances in antimicrobial therapy and supportive care have been achieved in the past few decades (Naito et al., 2013). Accumulating evidence proved that various factors including dysregulation of inflammatory/anti-inflammatory pathway and oxidant/antioxidant dysfunction contributed to the pathogenesis of ALI (Hu et al., 2016; Ding et al., 2017; Lu et al., 2017). Unfortunately, there are still no effective treatments that could significantly reduce lung injury.

Traditional Chinese medicine has been widely used by almost one–fifth of the world’s population since ancient times and is still acknowledged as an important source of pharmaceutical remedies (Chen et al., 2015). Chelerythrine (CH) is a kind of benzo[c] phenanthridine alkaloid, with pharmacological activities as antitumor, antibiosis and anti-inflammation, which is widely found in plant of Fumariaceae, Papaveraceae, Ranunculaceae and Rutaceae families (Niu et al., 2011; Pencikova et al., 2012; Lin et al., 2017). CH inhibited the release/production of exudates and prostaglandin E (2) mediated through cyclooxygenase-2 regulation (Niu et al., 2011). CH could exert its anti-inflammatory effect through the inhibition of 5-lipoxygenase, attenuation of the oxidative burst and suppression of the P2X7 receptor activity (Shemon et al., 2004; Vrba et al., 2008). However, few studies have demonstrated whether CH could protect against lipopolysaccharide (LPS)-induced ALI, and the underlying mechanism is also uncertain. This study revealed protective effects of CH on LPS-induced ALI both *in vivo* and *in vitro*.

## Materials and Methods

### Reagents

CH (pure: 99%) was provided by National Institutes for Food and Drug Control (Beijing, China). Myeloperoxidase (MPO), Malondialdehyde (MDA), Superoxide dismutase (SOD), and Wright–Giemsa staining kits were purchased from the Institute of Jiancheng Bioengineering (Nanjing, China). The enzyme-linked immunosorbent assay (ELISA) kits for determination of Tumor necrosis factor-alpha (TNF-α), interleukin 6 (IL-6), and interleukin 1β (IL-1β) were produced by Nanjing KeyGEN Biotech. CO., LTD (Nanjing, China). Antibodies against nuclear factor erythroid 2-related factor 2 (Nrf2) (#12721), heme oxygenase (HO-1) (#70081), cyclooxygenase2 (Cox2) (#12282), nuclear factor kappa-B (NF-κB) p65 (#8242), IκBα (#4814), Phospho-IκBα (#2859), and β-Actin (#3700) were purchased from Cell Signaling Technology (Beverly, MA, United States). Lamin B1 (ab133741) and inducible Nitric Oxide Synthase (iNOS) (ab3523) antibodies were purchased from Abcam (Cambridge, MA, United States). Dimethyl sulfoxide (DMSO) and LPS were obtained from Sigma-Aldrich (St. Louis, MO, United States). Protease Inhibitor Cocktail was obtained from Roche Technology (Basel, Switzerland). N-acetyl-L-cysteine (NAC) and 2′,7′-Dichlorofluorescin diacetate (DCFH-DA) were obtained from Beyotime Biotech (Nantong, China).

### Cell Culture

RAW264.7 cells were purchased from Shanghai Institute of Cell Biology (Shanghai, China) and cultured in RPMI 1640 (Gibco, Grand Island, NY, United States) supplemented with 10% fetal bovine serum (Gibco), 100 U/ml penicillin and 100 mg/ml streptomycin, and incubated under a humidified 5%(v/v) CO_2_ atmosphere at 37°C. Cells were treated with various concentrations of CH or 0.1% DMSO as control for 1 h, followed by 1 μg/ml LPS stimulation for 2 h. CH was dissolved in DMSO to a concentration of 2 mM (stock solution) and stored at –20°C. LPS was dissolved in H_2_O to a concentration of 1 mg/ml (stock solution) and stored at –20°C.

### Detection of Intracellular ROS

Cells were incubated at the indicated conditions with 10 μM 2′,7′-Dichlorofluorescin diacetate (DCFH-DA) at 37°C for 20 min, and then washed in PBS. Fluorescence were measured at 488 nm excitation and at 535 nm emission with a fluorescence microscope (Olympus, Japan). Results were expressed as relative to control DCF fluorescence.

### Animals

Animal welfare and experimental procedures were conducted in accordance with the Provision and General Recommendation of Chinese Experimental Animals Administration Legislation and were approved by Animal Ethics Committee of Nanjing University of Chinese Medicine (NZY-20170530).

A total of 50 female BALB/c mice (18–22 g), obtained from Jiangning Qinglongshan Animal Cultivation Farm (Nanjing, China), was maintained in an animal facility under standard laboratory conditions for 7 days prior to experiments. Then the animals were randomly assigned to one of five groups (*n* = 10 per group). The groups consisted of control group (saline), LPS-induced ALI group, CH treatment only group (CH, 10 mg/kg), CH treatment of LPS-induced ALI group (CH, 5 mg/kg), CH treatment of LPS-induced ALI group (CH, 10 mg/kg). CH treatments were carried out for 7 consecutive days prior to LPS challenge by gavage. After CH treatment, the mice were anesthetized 2% sodium pentobarbital (80 mg/kg, Sigma-Aldrich, St. Louis, MO, United States) by intraperitoneal injection. Then, LPS (5 mg/kg, 0.9% saline) was administrated intratracheally to induce ALI. Control group mice were given equal volume 0.9% saline. Six hours later, all animals were sacrificed. Then, serum, bronchoalveolar lavage fluid (BALF, see below) and lung tissue samples were collected and stored.

### Collection of BALF and Cell Counting

The bronchoalveolar lavage (*n* = 10) was harvested three times through a tracheal cannula with 0.5 ml (total volume 1.5 ml) of autoclaved phosphate buffer saline (PBS) to obtain the BALF. The total leukocyte count was determined using a hemocytometer. BALF samples were centrifuged at 1000 g for 5 min at 4°C, the supernatants were stored in –80°C for the further tests and the pellet was resuspended in 100 μl of PBS, stained with Wright-Giemsa staining (Nanjing Jiancheng Bioengineering Institute, Nanjing, China) on the slides. The slides were quantified for neutrophils by counting a total of 200 cells.

### Lung Wet-to-Dry Weight Ratios

The right lungs were excised at the end of the experiment. The trachea and esophagus were separated from the lungs by blunt dissection, and wet weight was determined immediately. Subsequently, the lungs were incubated at 60°C for 48 h to remove all moisture. Then the dry weight was measured and the ratio of wet-to-dry was calculated.

### Analysis of MPO, SOD Activity, and MDA Content in Lung Tissues

The lung tissues from left upper lobe were homogenized in normal saline. The homogenates were centrifuged (12,000 rpm, 4°C, 20 min), and the supernatants were collected for subsequently measurements. MPO, MDA, and SOD levels in the tissues were determined using test kits purchased from Nanjing Jiancheng Bioengineering Institute (Nanjing, China). All procedures were according to the manufacturers.

### Histological Assessment

The lung tissues from left lower lobe were fixed in 4% paraformaldehyde for 48 h. Then the samples were dehydrated in graded alcohol and embedded in paraffin wax. After that, the ematoxylin and eosin (H&E) staining was performed according to the protocol (Fan et al., 2014). Samples were then examined with a microscope (Olympus, Japan).

### Reverse Transcription and Quantitative PCR (Q-PCR)

Total RNA was isolated from cells as described (Fan et al., 2017). Quantitative PCR was carried out with the ABI Prism 7000 sequence detection system (Applied Biosystems, Foster City, CA, United States) using SYBR Green I dye (Biotium, Inc., Hayward, CA, United States). The primer sequences used were as follows (5′–3′): *Tnf* (NM_013693.3) sense, CTTCTCATTCCTGCT\TGTG and antisense ACTTGGTGGTTTGCTACG; *Il1b* (NM_008361.4) sense, CCTGGGCTGTCCTGATGAGAG and antisense TCCACGGGAAAGACACAGGTA; *Il6* (NM_031168.2) sense, CTGCAAGAGACTTCCATCCAG and antisense AGTGGTATAGACAGGTCTGTTGG; *GAPDH* (NM_001289726.1) sense, AATGGATTTGGACGCATTGGT and antisense TTTGCACTGGTACGTGTTGAT.

### Cell Viability Assay

Cell viability was assessed with Methylthiazoletetrazolium (MTT) assay. Briefly, the RAW264.7 cells were plated at a density of 3.0 × 10^3^ cells/well in 96-well plates and incubated at 37°C for 24 h. The cells were treated with different concentrations of CH for another 24 h. Then 20 μl MTT (4 mg/ml in PBS) was added to each well and incubated at 37°C for 4 h. After removing incubation medium, the purple formazan crystals were dissolved in 200 μl of DMSO for 5 min. The absorbance values were measured at 570 nm using a microplate spectrophotometer (Tecan, Switzerland).

### Cytokine Assay

The levels of TNF-α, IL-6, and IL-1β in BALF, serum and in the supernatant of RAW264.7 cells were measured using ELISA kits according to the manufacturer’s instructions provided by Nanjing KeyGEN Biotech. CO., LTD (Nanjing, China). The optical density (OD) of each well was detected with a microplate spectrophotometer.

### Western Blotting

The total protein of cells or lung tissues from left upper lobe were extracted and immunoblots were performed as described (Fan et al., 2017). Nuclear proteins were prepared using cytoplasmic and nuclear protein extraction kit (KeyGEN, Nanjing, China). All of the protein fractions were stored at –80°C until use.

### Small Interfering RNA (siRNA) Transfection

Nrf2 siRNA sequence and Luciferase siRNA used in RAW264.7 cells were purchased from (Thermo Fisher Scientific, Hudson, NH, United States) Cells were transfected with luciferase siRNA or Nrf2 siRNA using Lipofectamine 2000 (Life Technologies, Carlsbad, CA, United States) according to the manufacturer’s instructions (Fan et al., 2014). Then, the cells were treated with 2 μM CH or 0.1% DMSO as control for 1 h, followed by LPS stimulation for 2 h.

### Statistical Analysis

The results were expressed as means ± SD and analyzed by one-way ANOVA and Tukey’s *post hoc* tests were applied when there are more than two groups in the independent variable. *P*-value < 0.05 is regarded as statistically significant.

## Results

### Effects of CH on LPS-Induced Lung Injury in Mice

**FIGURE 1:**
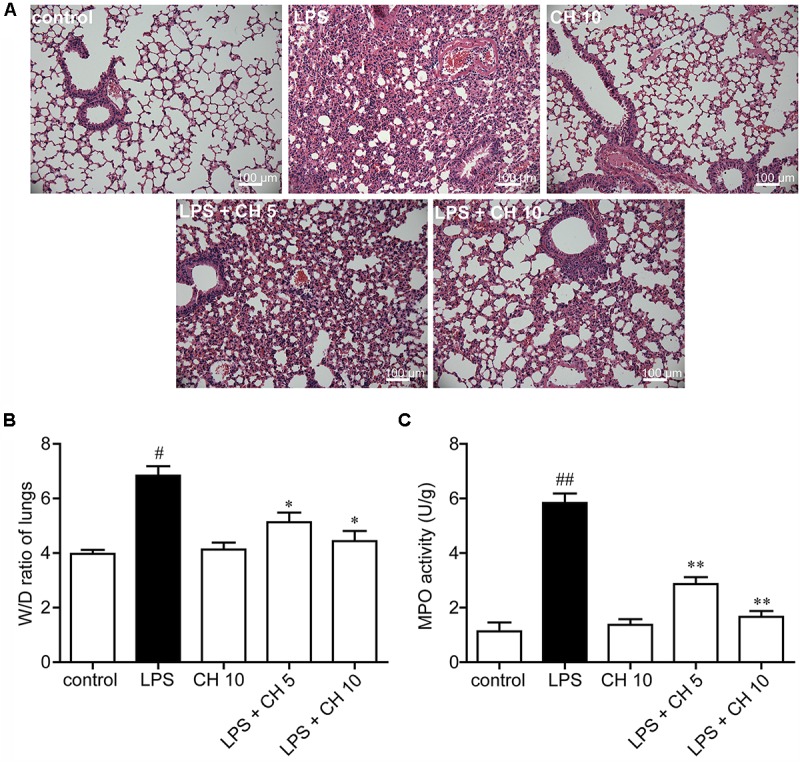
Effects of CH on LPS-induced lung injury in mice. **(A)** Representative histological images of lung tissues harvested from mice following LPS challenge showing the effect of CH. Tissues were stained with H&E (Scale bar = 100 μm). **(B)** Effects of CH on Lung wet/dry (W/D) ratio. **(C)** Effects of CH on MPO activity. Values were expressed as mean ± SD; *n* = 10 mice per group. ^#^*p* < 0.05, ^##^*p* < 0.01 versus control group. ^∗^*p* < 0.05, ^∗∗^*p* < 0.01 versus LPS group.

As shown in **Figure 1A**, the lungs of mice demonstrated a large number of neutrophil infiltration around the pulmonary vessel and airway, thickening of alveolar walls, and disruption of endothelial and epithelial integrity after intratracheally stimulated with LPS. On the contrary, CH treatment groups significantly improved the lung injury induced by LPS. Further evidence of LPS-induced structural deficits in lungs is increased wet/dry weight ratio (*p* < 0.05, **Figure 1B**), and increased MPO activity (*p* < 0.01, **Figure 1C**). The results indicate that CH could ameliorate the condition of pathological inflammation in pulmonary tissues of ALI induced by LPS.

### Effects of CH on Pro-inflammatory Cytokines in BALF and Serum

**FIGURE 2:**
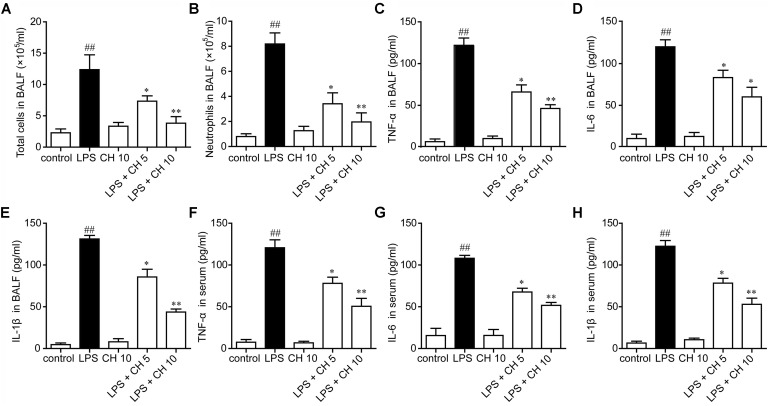
Effects of CH on pro-inflammatory cytokines in BALF and serum. CH reduced the number of total cells **(A)** and neutrophils **(B)** in BALF following LPS challenge. **(D–G)** CH inhibited LPS-induced inflammatory cytokines, TNF-α **(C,F)**, IL-6 **(D,G)**, and IL-1β **(E,H)** in BALF and serum. Values were expressed as mean ± SD; *n* = 10 mice per group. ^##^*p* < 0.01 versus control group. ^∗^*p* < 0.05, ^∗∗^*p* < 0.01 versus LPS group.

In our study, we found that LPS significantly increased the neutrophils in BALF, while pretreatment with CH significantly reduced the neutrophils in BALF (*p* < 0.01, **Figures 2A,B**). Inflammatory cytokines were involved in the initiation, amplification, perpetuation of inflammatory cascade in LPS-induced ALI (Stormann et al., 2017). Next, we detected the cytokine levels in BALF and serum. The results showed that LPS significantly increased the TNF-α, IL-6, and IL-1β in BALF and serum compared with those in the control group. Moreover, CH pretreatment dose-dependently prevented LPS-mediated increases of these cytokines (*p* < 0.01, **Figures 2C–H**).

### CH Attenuates Lung Oxidative Stress in LPS-Induced Lung Injury in Mice

**FIGURE 3:**
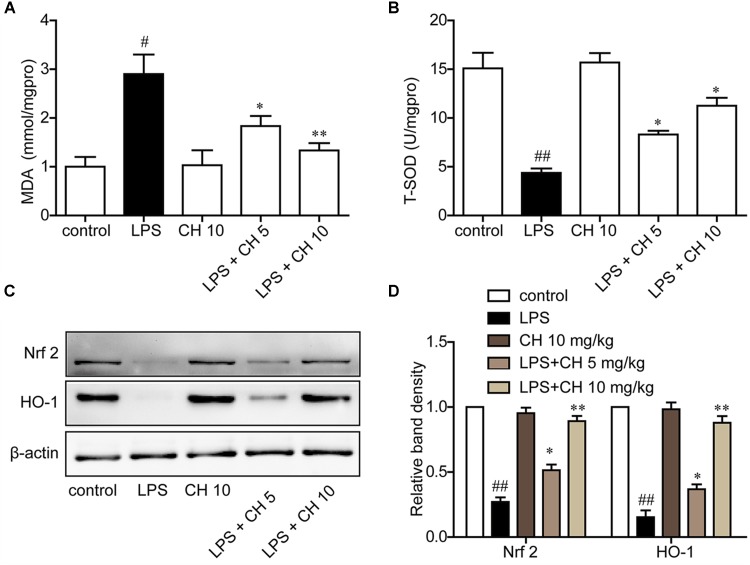
CH attenuated lung oxidative stress in LPS-induced lung injury in mice. **(A)** Production of MDA in lung tissue. **(B)** The activity of T-SOD in lung tissue. **(C,D)** Immunoblots against Nrf2, HO-1, and β-actin were detected. β-actin was taken as control. Blots are representative of the five groups. Values were expressed as mean ± SD. ^#^*p* < 0.05, ^##^*p* < 0.01 versus control group. ^∗^*p* < 0.05, ^∗∗^*p* < 0.01 versus LPS group.

LPS significantly increased MDA content and decreased T-SOD activity, while CH pretreatment markedly reversed these enzyme activity (*p* < 0.01, **Figures 3A,B**). Western blot analysis showed that the expression of antioxidant responsive protein which including Nrf2 and HO-1 were decreased markedly in LPS-induced ALI. Treatment with CH dose-dependently activated Nrf2 and HO-1 (*p* < 0.01, **Figures 3C,D**).

### Effects of CH on Pro-inflammatory Cytokines in RAW264.7 Cells

**FIGURE 4:**
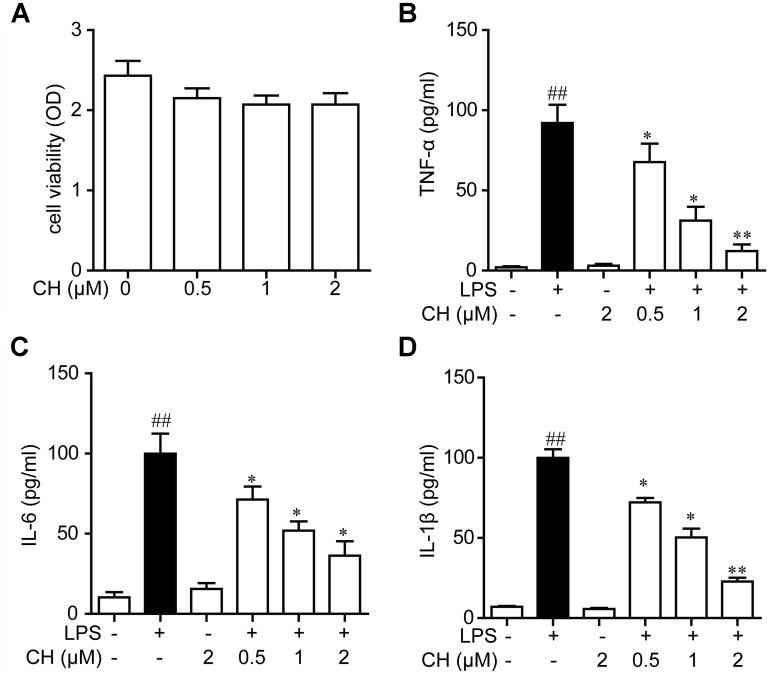
Effects of CH on pro-inflammatory cytokines in RAW264.7 cells culture supernatants. **(A)** RAW264.7 cells were incubated with CH at various concentrations for 24 h. The cell viability was determined by MTT assay. Values were expressed as mean ± SD of three independent experiments. **(B–D)** RAW264.7 cells were pretreated with CH at 0.5, 1, or 2 μM for 1 h and then treated with LPS at 1 μg/ml for 2 h. DMSO was used as vehicle control for CH. TNF-α **(B)**, IL-6 **(C)**, and IL-1β **(D)** cytokine levels in the culture medium were measured by ELISA. Values were expressed as mean ± SD of three independent experiments. ^##^*p* < 0.01 versus control group. ^∗^*p* < 0.05, ^∗∗^*p* < 0.01 versus LPS group.

Next, we examined the effects of CH on pro-inflammatory cytokines in RAW264.7 cells using MTT assay. As shown in **Figure 4A**, CH did not display any cellular toxicity at 0.5, 1, and 2 μM. These results proved that the inhibitory effect caused by CH treatment was not due to its cytotoxity. To further confirm the anti-inflammatory activity of CH *in vitro*, the levels of TNF-α, IL-6, and IL-1β in cell supernatant was detected. Consistent with the result *in vivo*, LPS significantly increased the TNF-α, IL-6, and IL-1β in RAW264.7 cells, while CH pretreatment dose-dependently prevented LPS-mediated increases of these cytokines (*p* < 0.01, **Figures 4B–D**).

### Effects of CH on the NF-κB Pathway Activation in LPS-Stimulated RAW264.7 Cells

**FIGURE 5:**
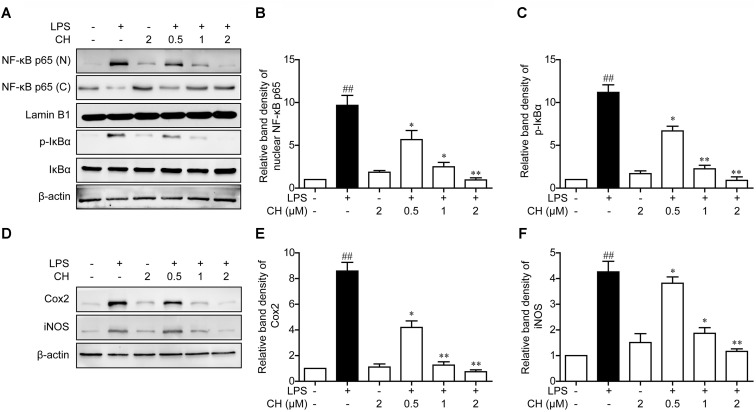
Effects of CH on the NF-κB pathway activation in LPS-stimulated RAW264.7 cells. RAW264.7 cells were pretreated with CH at 0.5, 1, or 2 μM for 1 h and then treated with LPS at 1 μg/ml for 2 h. DMSO was used as vehicle control for CH. **(A–C)** Expressions of NF-κB in cytosol and nuclear **(B)**, phosphorylated or total forms of IκBα **(C)** were measured by western blot. β-actin and Lamin B1 was taken as control. **(D–F)** Immunoblots against Cox-2 **(E)**, iNOS **(F)** and β-actin from cell lysates of RAW264.7 were detected. Values were expressed as mean ± SD of three independent experiments. ^##^*p* < 0.01 versus control group. ^∗^*p* < 0.05, ^∗∗^*p* < 0.01 versus LPS group.

It is well known that NF-κB is a key regulator involved in inflammatory process. NF-κB is an important transcription factor involved in regulating inflammatory and immune responses. The activation of NF-κB owes to its phosphorylation and degradation of IκB which is combined with NFκB, leading to the translocation of NF-κB into the nucleus to induce the expression of proinflammatory mediators (Pace et al., 2016). To further explore the anti-inflammatory mechanisms of CH, we assessed the activation of the NF-κB pathway in LPS-stimulated RAW264.7 cells. As revealed in **Figure 5A**, LPS exposure led to the translocation of the NF-κB p65 from the cytosol to the nucleus. However, significant reduction of nuclear p65 protein was observed in RAW264.7 cells pretreatment with CH in a dose-dependent manner (*p* < 0.01, **Figure 5A**). Meanwhile, the expression of downstream targets of NF-κB, iNOS, and Cox2, were dose-dependently reduced by CH in LPS treated RAW264.7 cells (*p* < 0.01, **Figure 5D**). Taken together, these results indicate that CH inhibits LPS-induced inflammatory responses by attenuating the release of pro-inflammatory cytokines through the inhibition of NF-κB activation.

### Effects of CH on the LPS-Induced Stimulation of Reactive Oxygen Species (ROS)

**FIGURE 6:**
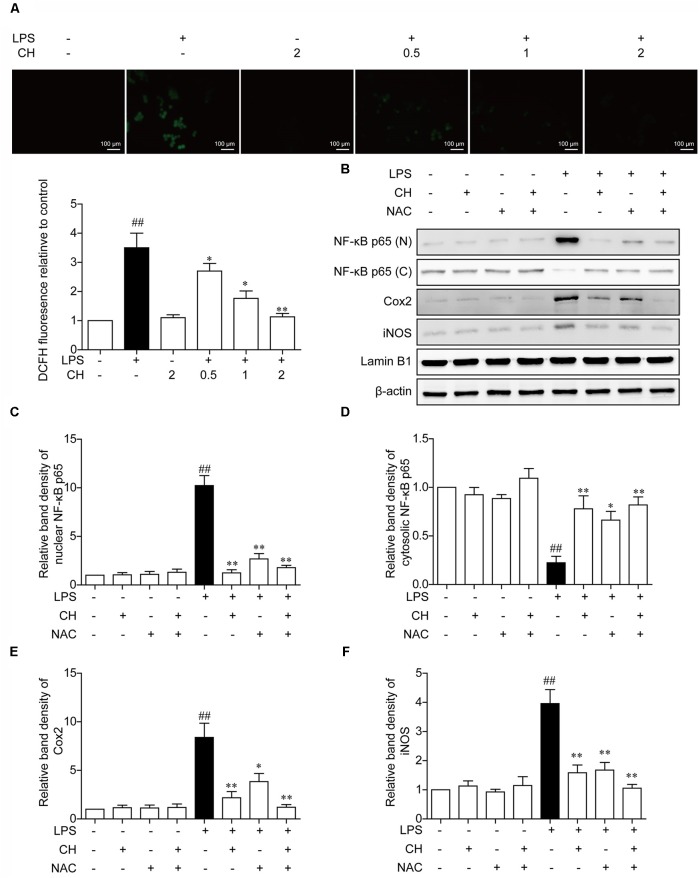
Effects of CH on the production of ROS in LPS-stimulated RAW264.7 cells. **(A)** RAW264.7 cells were pretreated with CH at 0.5, 1, or 2 μM for 1 h and then treated with LPS at 1 μg/ml for 2 h. DMSO was used as vehicle control for CH. Intracellular ROS induced by LPS was measured using respective fluorescent dyes DCFH-DA. Values were expressed as mean ± SD of three independent experiments. ^##^*p* < 0.01 versus control group. ^∗^*p* < 0.05, ^∗∗^*p* < 0.01 versus LPS group. **(B–F)** RAW264.7 cells were pretreated with 2 μM CH for 1 h or 5 mM NAC for 2 h, and then treated with LPS at 1 μg/ml for 2 h. DMSO was used as vehicle control for CH. Immunoblots against NF-κB in nuclear **(C)** and cytosol **(D)**, Cox-2 **(E)**, and iNOS **(F)** from cell lysates of RAW264.7 were detected. β-actin and Lamin B1 was taken as control. Values were expressed as mean ± SD of three independent experiments. ^##^*p* < 0.01 versus control group. ^∗^*p* < 0.05, ^∗∗^*p* < 0.01 versus LPS group.

*In vivo* experiments also implied that CH may possess a potential anti-antioxidant effect. To confirm this hypothesis, Effects of CH on the LPS -induced stimulation of reactive oxygen species (ROS) was detected by DCFH-DA. As shown in **Figure 6A**, LPS induced ROS rise was significantly restrained by CH in a dose-dependent manner (*p* < 0.01, **Figure 6A**). To further investigate the role of ROS in inflammation induced by LPS, NAC, an active oxygen inhibitor, which inhibits the production of ROS, was used. Compared with the LPS-treated group, pretreatment with NAC strongly dampened NF-κB translocation and reduced the expression of downstream targets of NF-κB, iNOS, and Cox2 (*p* < 0.01, **Figures 6B–F**), which indicated that NAC can dampen NF-κB translocation by inhibiting the LPS-induced stimulation of ROS.

### CH Nrf2-Dependently Regulates NF-κB Pathway Activation in LPS-Stimulated RAW264.7 Cells

**FIGURE 7:**
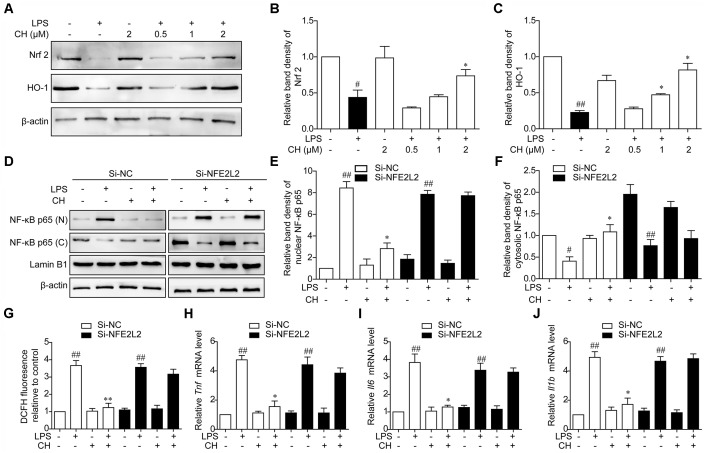
CH Nrf2-dependently regulates NF-κB pathway activation in LPS-stimulated RAW264.7 cells. **(A–C)** RAW264.7 cells were pretreated with CH at 0.5, 1, or 2 μM for 1 h and then treated with LPS at 1 μg/ml for 2 h. DMSO was used as vehicle control for CH. Immunoblots against Nrf2 **(B)**, HO-1 **(C)**, and β-actin were detected. β-actin was taken as control. Blots are representative of three independent experiments. Values were expressed as mean ± SD of three independent experiments. ^#^*p* < 0.05, ^##^*p* < 0.01 versus control group. ^∗^*p* < 0.05, versus LPS group. **(D–J)** RAW264.7 cells were transfected with Luc siRNA (Si-NC) or Nrf2 siRNA (Si-NFE2L2) for 24 h, followed by incubation with 2 μM CH for 1 h and then treated with LPS at 1 μg/ml for 2 h. **(D–F)** The expressions of NF-κB in nuclear **(E)** and cytosol **(F)** were measured by western blot. β-actin and Lamin B1 was taken as control. Values were expressed as mean ± SD of three independent experiments. ^#^*p* < 0.05, ^##^*p* < 0.01 versus control group. ^∗^*p* < 0.05, versus LPS group. **(G)** Intracellular ROS induced by LPS was measured using respective fluorescent dyes DCFH-DA. Values were expressed as mean ± SD of three independent experiments. ^##^*p* < 0.01 versus control group. ^∗∗^*p* < 0.01 versus LPS group. **(H–J)** Representative mRNA levels of *Tnf* **(H)**, *Il6* **(I)**, and *Il1b* **(J)**. GAPDH was taken as control. Values were expressed as mean ± SD of three independent experiments. ^##^*p* < 0.01, versus control group. ^∗^*p* < 0.05, versus LPS group.

To assess the role of ROS in NF-κB translocation dampened by CH, the expression of antioxidant responsive protein including Nrf2 and HO-1 was detected. Consistent with the results *in vivo*, treatment with CH dose-dependently activated Nrf2 and HO-1 decreased by LPS in RAW264.7 cells (*p* < 0.05, **Figure 7A**). Furthermore, to determine whether activation of Nrf2 contributes to effect of CH on lung protection, Nrf2 was knocked down in RAW264.7 cells using Nrf2 siRNA (Si-NFE2L2). As shown in **Figure 7D**, while CH reduced the translocation of the NF-κB p65 from the cytosol to the nucleus in LPS-stimulated RAW264.7 cells, its modulation on NF-κB p65 translocation was strongly diminished when Nrf2 was knocked down (*p* < 0.01, **Figure 7D**). Meanwhile, CH pretreatment didn’t show any effect on ROS production in LPS-stimulated RAW264.7 cells transfected with Nrf2 siRNA (*p* < 0.01, **Figure 7G**). Furthermore, LPS-induced increases in TNF-α, IL-6, and IL-1β production weren’t reduced by CH in RAW264.7 cells transfected with Nrf2 siRNA (*p* < 0.01, **Figures 7H–J**). These results strongly indicate that Nrf2 is essential for CH’s protection against LPS induced ALI.

## Discussion

Acute lung injury is characterized by lung edema, extensive neutrophil infiltration, disruption of endothelial and epithelial integrity and release of pro-inflammatory mediators (Yu et al., 2014). The proinflammatory path is triggered in major parts by IL-1β and TNF-α. These cytokines are released during early phases and cause the release of further proinflammatory cytokines-among them IL-6 (Stormann et al., 2017). Despite being considered a pro-inflammatory cytokine, IL-6 could also exert anti-inflammatory effects (Starkie et al., 2003; Menghini et al., 2014). Chelerythrine, a specific inhibitor of protein kinase C that interacts with the catalytic subunit, has been shown to decrease the level of IL-6 mRNA and CCL-2 expression in LPS stimulated THP-1 cells (Pencikova et al., 2012). In this study, we found that CH pretreatment markedly inhibited pulmonary edema and the neutrophil infiltration in LPS induced ALI. Furthermore, TNF-α, IL-6, and IL-1β increased evidently in BALF and cell supernatant after LPS exposure. As expected, CH pretreatment significantly decreased the TNF-α, IL-6, and IL-1β in BALF and in cell supernatant.

NF-κB plays an important role in regulating of proinflammatory mediators in ALI (Zhu et al., 2017; Zhang et al., 2018). NF-κB is activated by various stimuli such as cytokines, ROS, bacterial or viral products (Zhao M.X. et al., 2017). Activated NF-κB translocates into the nucleus where it triggers the transcription of specific genes such as TNF-α, IL-1β, and IL-6 (Tang et al., 2017). NF-κB binding sequences have also been identified in proinflammatory genes such as iNOS and COX-2 (Zhang et al., 2018). The protective effects of CH on LPS-induced endotoxic shock can be attributed to attenuating inflammatory cytokines and inhibition of the expression of NF-κB (Niu et al., 2014). In our study, we showed that CH inhibited LPS-induced NF-κB activation in RAW264.7 cells. Moreover, iNOS, COX-2 were also suppressed by CH.

Exposed to high concentration of oxygen for over time will suppress the ability to scavenge excessive ROS, conducing to various pulmonary dysfunction, such as membrane injury, intracellular edema and lipid peroxidation (Yu et al., 2015). The most important source of oxidative stress in ALI is attributed to the consumption of oxygen that results in the production of superoxide anions during neutrophil recruitment (Tsai et al., 2017). Previous investigators displayed that MDA and SOD were closely associated with the inflammatory pathogenesis of LPS-induced ALI (Jing et al., 2015). MDA is one of the major reliable index of lipid peroxidation and has been reported to play a critical role in the LPS-induced ALI (Kumari et al., 2015). MDA is an important marker of tissue damage, both at central and peripheral level, and natural compounds exerting protective effects are able to blunt MDA upregulated levels induce by oxidant and inflammatory stimuli, including LPS (Ferrante et al., 2017; Locatelli et al., 2018; Menghini et al., 2018). Evidence has emerged indicated that SOD is involved in the mediation of transcription factors which govern the generations of inflammatory cytokines (Ward, 2010). The present study showed that CH remarkedly reduced oxidative burden during the inflammatory response to LPS both *in vivo* and *in vitro*.

Endogenous antioxidants cope with the oxidative burden and limit potential toxicity of ROS. Nrf2 is critical in cytoprotection by inducing expression of antioxidant and detoxifying enzymes and proteins via its binding to the cis-acting antioxidant response element (ARE) (Zhao H. et al., 2017). Numerous *in vitro* and *in vivo* studies have demonstrated the importance of Nrf2 activation to decrease oxidative stress and inflammation in conditions such as ALI (Cho et al., 2015). The Nrf2 pathway could support TNF-induced NF-kB-mediated survival signaling (Blaser et al., 2016). We showed that CH activated Nrf2 pathway decreased by LPS both *in vitro* and *in vivo*. Based on our results, we expected the role of Nrf2 in cytoprotect effects of CH against LPS-induced ALI. Therefore, Knockdown of Nrf2 gene was performed. We assumed that knockdown of Nrf2 diminished CH-mediated protective effects in ALI.

## Conclusion

**FIGURE 8:**
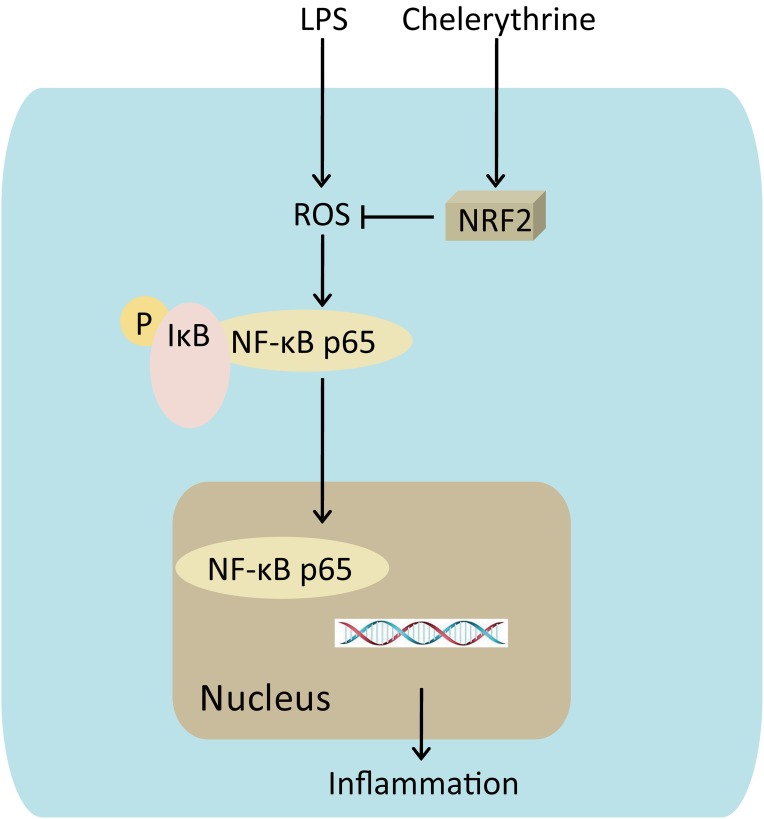
Mechanism for CH’s effects on LPS induced ALI. CH protection against LPS induced ALI through Nrf2 activation, knocking down of Nrf2 leading to the reduction of nuclear translocation of the NF-κB p65, which triggers inflammation.

These results of the present study revealed that CH exhibited protective effects on LPS-induced ALI *in vivo* and *in vitro* by attenuating the inflammatory response through NF-κB pathway mediated by Nrf2 (**Figure 8**). Therefore, CH may be considered as a potential agent for the treatment of ALI in the future.

## Author Contributions

LF and HW conceived and designed the study. LF, YF, LLiu, WT, XS, and YD acquired, analyzed, and/or interpreted the data. LF, YF, LLi, SZ, and HW drafted/revised the work for intellectual content and context. HW contributed to final approval and overall responsibility for the published work.

## Conflict of Interest Statement

The authors declare that the research was conducted in the absence of any commercial or financial relationships that could be construed as a potential conflict of interest.
